# Biochemical and Biomechanical Properties of Scaffold-Free Hyaline Cartilage Generated Under Dynamic Conditions

**DOI:** 10.3390/ijms26104719

**Published:** 2025-05-15

**Authors:** Fernando P. S. Guastaldi, David M. Kostyra, Nichaluk Leartprapun, Seemantini Nadkarni, Mark A. Randolph, Robert W. Redmond

**Affiliations:** 1 Division of Oral and Maxillofacial Surgery, Department of Surgery, Harvard School of Dental Medicine, Massachusetts General Hospital, Boston, MA 02114, USA; fguastaldi@mgh.harvard.edu; 2 Wellman Center for Photomedicine, Harvard Medical School, Massachusetts General Hospital, Boston, MA 02114, USA; dmk3mchs@gmail.com (D.M.K.); nleartprapun@mgh.harvard.edu (N.L.); snadkarni@mgh.harvard.edu (S.N.); 3 Plastic Surgery Research Laboratory, Department of Surgery, Harvard Medical School, Massachusetts General Hospital, Boston, MA 02114, USA; marandolph@mgh.harvard.edu

**Keywords:** articular cartilage, cartilage regeneration, chondrocytes, tissue engineering, dynamic self-regenerating cartilage, dSRC, shear

## Abstract

Developing a functional tissue-engineered articular cartilage remains a challenge to improving clinical treatment of cartilage injury and joint-related degenerative disease. The dynamic self-regenerating cartilage (dSRC) approach presented here encourages autologous chondrocytes to generate their own matrix rather than imposing a matrix upon them. dSRC constructs were grown for 12 weeks under hypoxic conditions in reciprocating motion. Biochemical composition was evaluated, specifically water, collagen, and proteoglycan content. Speckle rHEologicAl micRoscopy (SHEAR) was utilized for spatially resolved evaluation of the shear modulus in engineered cartilage. Histological and immunohistochemical analyses of dSRC were also performed. The maturation of the dSRC matrix results in collagen and glycosaminoglycan (GAG) levels around 50% of those in native cartilage. SHEAR images demonstrate an increase in shear modulus of the matrix to ~20% that of native cartilage after 12 weeks. Histological support for excellent collagen and GAG production was evident, and immunohistochemistry showed a high preference for hyaline-like type II collagen in the neomatrix. A decrease in chondrocyte density occurred from an initial hypercellular matrix to that approaching native cartilage by 12 weeks. While this maturation of dSRC in vitro should not be construed as an absolute prediction of in vivo performance, these results are encouraging, representing a potential new cartilage repair and regeneration approach.

## 1. Introduction

Focal or post-traumatic injury to articular cartilage in joints can result from blunt trauma, occupational strain, sporting and recreational activity, and overuse [1,2,3]. Treatment is still mainly limited to palliative care [4]. Current surgical repair options for the knee include microfracture, osteochondral autograft, and autologous chondrocyte implantation (ACI) [5] with or without a scaffold carrier [4,6,7]. However, while improvement has been reported [5,8,9], some of these treatments typically result in the formation of nondurable fibrocartilage and can cause complications due to donor-site morbidity. There is clearly an unmet need in medicine for an effective treatment for cartilage injury that would generate normal hyaline cartilage in the defect to provide functional recovery and prevent advanced osteoarthritis (OA) in the long term [4,7,10].

Unsatisfactory outcomes from current cartilage repair modalities have stimulated the development of scaffold- and cell-based regenerative approaches to articular cartilage repair [6] with the overall goal of developing regenerative strategies that reproduce the biomechanical characteristics of native hyaline cartilage. Several natural and synthetic materials have been tested [11], including polyethylene glycol (PEG) [12], hyaluronic acid [13], collagen [14], and silk [15] hydrogels and biphasic osteochondral [9,16] matrices, demonstrating highly tunable mechanical characteristics and the ability to degrade over time [17,18]. However, these scaffold materials lack the structure and organization characteristics of native hyaline cartilage and can cause an immune response or foreign body reaction in vivo. Furthermore, scaffolds may inhibit contiguous hyaline cartilage after implantation in terms of biochemical composition, biomechanical behavior, and integration with host tissue. New regenerative approaches to cartilage regeneration for articular repair must overcome these challenges [4,7]. Scaffold-free generation of cartilage could avoid these problems. Still, most reported strategies employ a monolayer culture of chondrocytes [19], which can lead to dedifferentiation of the cells and poor quality of the cartilage matrix.

Our group has previously demonstrated that autologous swine chondrocytes, cultured in dynamic reciprocating motion, self-generate a robust hyaline-like articular cartilage matrix that may be capable of addressing the challenges in biomechanical, biochemical, and structural properties of previous regenerative approaches. In an in vivo swine pilot study, Meppelink et al. [20] showed that this dynamic self-regenerating cartilage (dSRC) is strongly integrated with native cartilage, generating near-contiguous cartilage in knee chondral defects without evidence of fibrocartilage. In a recent in vitro and ex vivo study, we showed that the self-generated cartilage showed histologic evidence of hyaline-like composition and that the dSRC tissue matures with time toward hyaline cartilage [21].

While histologic evidence is valuable, a quantitative assessment of biochemical composition and biomechanical properties is essential to evaluate the development of engineered cartilage. In this study, a new imaging methodology, Speckle rHEologicAl micRoscopy (SHEAR), was employed to assess the shear modulus of native and engineered cartilage [22,23]. Current methods for mechanical testing of articular cartilage include compression, lubrication, tension, nanoindentation, and integration [24]. Rheometry is the established conventional method for measuring bulk shear mechanical properties, but it has significant limitations for mapping biomechanical properties in situ [25]. In contrast, SHEAR supports high-resolution mapping of shear viscoelastic modulus in whole tissue or tissue-engineered scaffolds in a noncontact fashion without deforming or manipulating the sample. In contrast, standard mechanical rheometry measures only bulk properties, which require mechanical deformation of the specimen and cannot be performed in situ. SHEAR can also resolve microscale spatial variations in shear modulus within a heterogeneous sample. This study uses SHEAR to evaluate the biomechanical properties in engineered cartilage, which were then correlated with the biochemical composition of dSRC as a function of maturation time, compared to native hyaline cartilage.

The relative amounts of the major constituents, water, collagen, and glycosaminoglycans (GAGs), were measured quantitatively, as they comprise the bulk of the extracellular matrix of native cartilage and give it its characteristic mechanical properties. The magnitude of the shear viscoelastic modulus was obtained via SHEAR and compared to parallel-plate shear rheometry, as described by Hajjarian et al. [26] and Hajjarian and Nadkarni [22]. Finally, histological and immunohistochemical analyses enabled elucidation of the structure and composition of dSRC as it matures over time in culture compared to native cartilage.

## 2. Results

### 2.1. dSRC Composition

**Figure 1:**
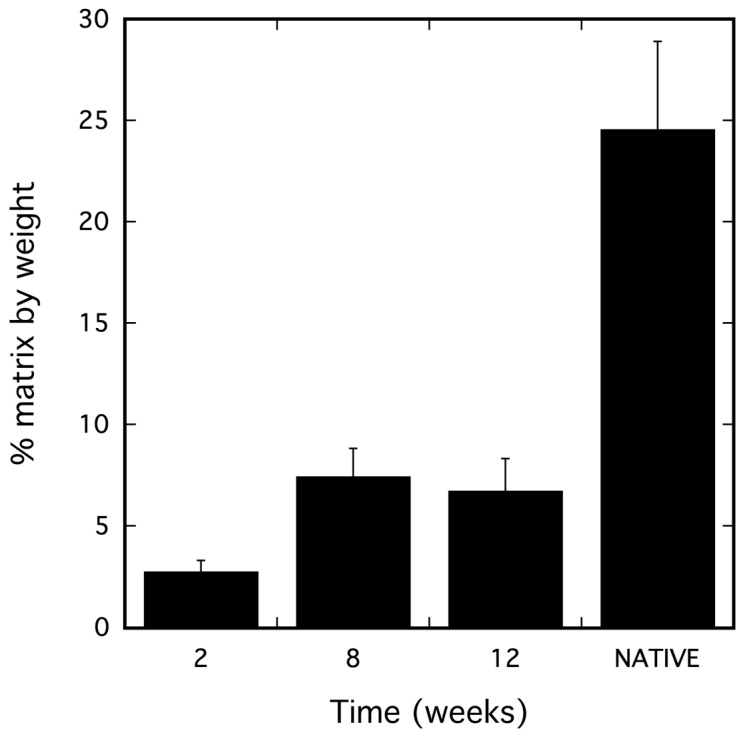
Percent matrix (dry weight/wet weight) for dSRC samples as a function of time and for native cartilage.

The percentage of the matrix in the dSRC samples and in native cartilage was calculated from pre- and post-lyophilization weights (the remaining weight is water) and illustrated in Figure 1. Quantitatively, the amount of GAG found in the 2-, 8-, and 12-week samples was 0.198 ± 0.031, 0.282 ± 0.014, and 0.231 ± 0.029 mg/mg of dry sample, respectively. Expressed as a percentage of the amount of GAG found in native swine articular cartilage (0.415 ± 0.019 mg/mg dry sample), the 2-, 8-, and 12-week values were 48%, 68%, and 56%, respectively. The amount of collagen in 2-, 8-, and 12-week samples was 0.111 ± 0.014, 0.120 ± 0.015, and 0.210 ± 0.013 mg/mg, respectively. This corresponds to values of 30%, 32%, and 52%, respectively, when expressed as a percentage of the amount of collagen found in native swine articular cartilage (0.371 ± 0.034 mg/mg).

### 2.2. Biomechanical Analysis

**Figure 2:**
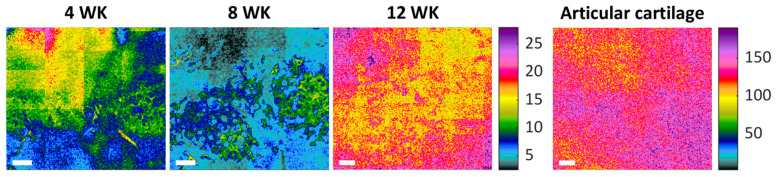
Representative shear modulus maps of native cartilage and dSRC matrix at 4, 8, and 12 weeks obtained with SHEAR. Color bars indicate the magnitude of shear modulus, |G*|, in kPa. All three dSRCs share the same false color scale for comparison as a function of maturation time. Native cartilage is displayed on a different false color scale due to its higher modulus magnitude. Scale bar: 250 μm.

**Figure 3:**
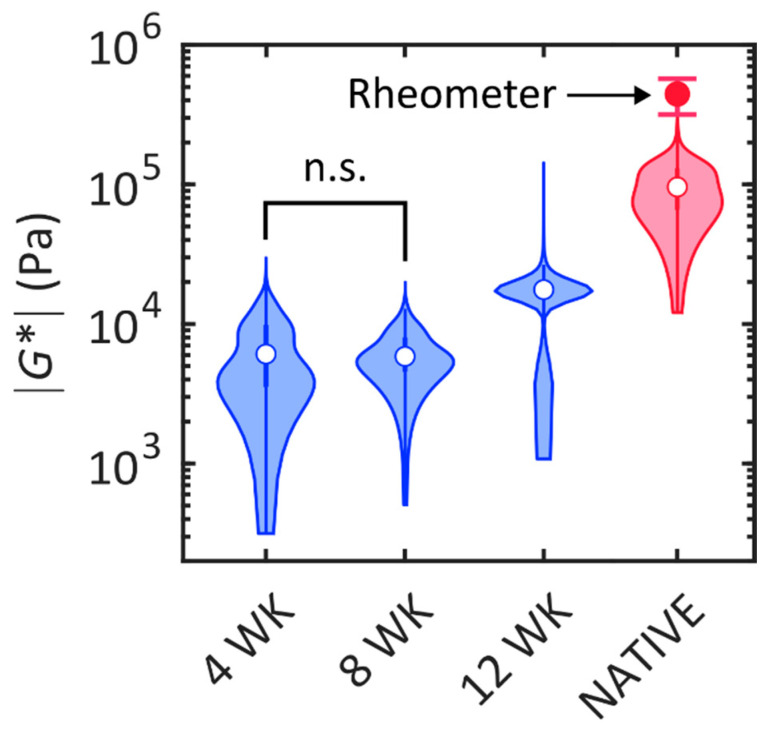
Shear modulus of native cartilage and dSRC matrix at 4, 8, and 12 weeks measured with SHEAR (violins) and a mechanical rheometer (red marker with error bars for native cartilage). For SHEAR, the violin shape represents the distribution of the magnitude of shear modulus, |G*|, over a region of 1.9 mm × 2.1 mm in 3 specimens for each category; the white marker indicates the median value. Nonparametric multiple comparisons with the Kruskal–Wallis test indicate a statistically significant difference (*p* < 0.0001) in the distribution of |G*| values between all pairs of categories except between dSRC at 4 and 8 weeks (n.s.: not significant). For the rheometer, the marker and error bar represent the mean ± standard deviation of 4 specimens.

The shear modulus of the dSRC samples was measured nondestructively with SHEAR in order to evaluate their mechanical performance relative to native cartilage. SHEAR is the only tool that permits high-resolution mapping of spatially varying mechanical properties of the arbitrarily shaped and fragile (at earlier stages of maturation) dSRC matrix in a noninvasive manner that preserves the samples for other analyses. A representative high-resolution spatial map of the magnitude of shear modulus, |G*|, for each dSRC maturation time point and native cartilage obtained by SHEAR is shown in Figure 2. These maps reveal the spatial variation in the mechanical firmness or “stiffness” within each of the dSRC samples and their mechanical maturation over time. The dSRC samples at week 4 and 8 exhibit localized regions with relatively higher |G*|, likely suggesting a nonuniform matrix deposition at earlier stages of maturation. By week 12, the |G*| map becomes more uniform with a higher average modulus value. However, the dSRC matrix remains substantially more compliant and relatively more heterogeneous than the native cartilage after 12 weeks of maturation. The distribution of |G*| in the dSRC matrix at each maturation time and the native cartilage is quantitatively compared in Figure 3. An increasing trend in |G*| is observed in the dSRC matrix as a function of maturation time, suggesting that the self-generated matrix becomes more firm and attains greater capacity to bear mechanical loads over time under dynamic culture. After 12 weeks of maturation, the dSRC matrix attains an average of 20% of the shear modulus exhibited by native cartilage using SHEAR. The values obtained for native cartilage by bulk rheometry are higher than those of SHEAR.

### 2.3. Histological Analysis of dSRC Maturation

**Figure 4:**
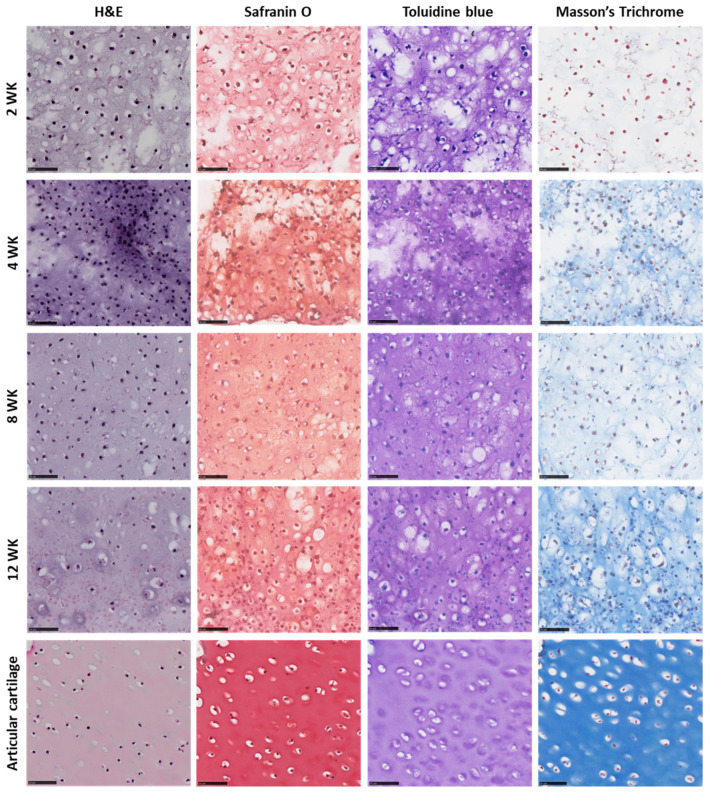
Representative histology of native cartilage and dSRC matrix at 2, 4, 8, and 12 weeks. Stains used were H&E (cell density), Safranin O (GAGs), Toluidine blue (collagen), and Masson’s Trichrome. Scale bar = 50 µm.

**Figure 5:**
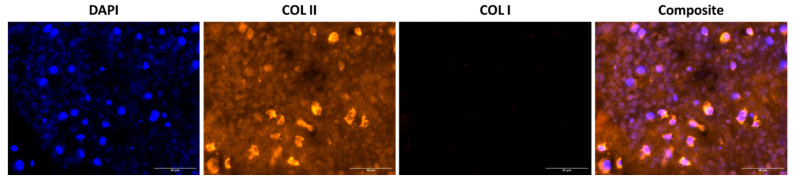
Representative images of the mature dSRC matrix at 12 weeks using DAPI (cell nuclei) and immunostaining for collagen type I and II. Scale bar: 50 µm.

Results demonstrate consistent formation of the dSRC matrix in vitro, after 2, 4, 8, and 12 weeks. The contiguous matrix is solid and robust. H&E staining shows a relatively high cell density in immature dSRC. Over time, chondrocytes appear more spread out, making the matrix less hypercellular (Figure 4). All dSRC groups demonstrated intense staining with Safranin O, indicating robust GAG production (Figure 4). Also, intense staining with Toluidine blue was observed in all dSRC samples, showing greater proteoglycan content (Figure 4). Intense staining with Masson’s Trichrome demonstrates an increased collagen content of the neomatrix. Immunofluorescence confirmed that the dSRC matrix, at 12 weeks, produces tissue with collagen type II in abundance over collagen type I, typical of hyaline cartilage (Figure 5).

## 3. Discussion

Our approach to engineering cartilage is unlike “standard” tissue engineering, whereby cells are seeded on scaffold matrices and grown in vitro for a period of time before in vivo implantation. Autologous chondrocytes self-generate a new native cartilage matrix (dynamic self-regenerating cartilage (dSRC)) on incubation in a relatively hypoxic environment under continuous dynamic motion. This self-assembling process is a potentially robust approach for rapidly engineering small and large cartilage constructs for implantation. Importantly, it does not involve a monolayer culture of chondrocytes, reducing the risk of cell dedifferentiation. Additionally, the dSRC implant would be placed in an immature state (e.g., after 2 weeks in culture), rather than a mature state [27,28], allowing the in situ physiological environment to “guide” the development of the neocartilage. Cartilage tissue is avascular and is generally considered a hypoxic environment. Thus, our approach mimics the native condition, allowing the chondrocytes to generate a matrix that is native to them rather than imposing a particular scaffold upon them.

In any approach for cartilage regeneration, it is important to identify the structure and biochemical composition of the matrix and its mechanical properties in relation to native healthy articular cartilage. While biochemical composition and histology are straightforward, mechanical performance is less so, especially in vivo. Two options for assessing tissue shear modulus are rheometry and SHEAR. SHEAR has been validated and applied in the biomechanical study of hydrogels [26], blood, and breast tumors [22]. It captures a spatial map of shear moduli, providing information on the mechanical heterogeneity in the microenvironments of each specimen. As conventional shear rheometry cannot assess the shear modulus of tissue-engineered constructs in a spatial or nondestructive manner [29], the micromechanical properties of dSRC and native swine articular cartilage were evaluated with SHEAR for the first time in this study. Compared to rheometry, SHEAR has the critical advantage of providing noncontact and noninvasive measurements, including arbitrarily shaped specimens such as the self-generated dSRC matrix. The ability to acquire such data suggests that SHEAR may provide a powerful platform for the future evaluation and progression of cartilage properties in diseased and repaired joints.

Considering the lack of physiological stimulation in this in vitro study, the value of ~20% shear modulus of native cartilage after 12 weeks in vitro is encouraging, as one may expect a greater value in the joint in vivo and a further increase longer after implantation. There is a considerable discrepancy between the shear modulus obtained from SHEAR and conventional rheometry, with the former resulting in an average modulus that is approximately 4x smaller than that obtained by rheometry. However, this may be explained by the fact that the shear modulus of native cartilage increases considerably with depth from the cartilage surface [30]. Rheometry provides a bulk measurement of the sample’s thickness (1 mm), including the stiffer tissue at greater depths. In contrast, in the current configuration, SHEAR predominantly probes the superficial depths where the modulus is lower. A strong correlation between SHEAR and conventional rheometry has been established in more homogeneous assorted hydrogel constructs and breast tissue specimens [26,31].

While the biomechanical properties of the neomatrix are crucial to the in situ performance of the construct, the biochemical composition and structure of the neocartilage are also important to the outcome. The dSRC tissue becomes stiffer with time in culture and exhibits increasing collagen and GAG content, with values >50% of native cartilage. An interesting feature of dSRC maturation is the obvious decrease in chondrocyte density over time as the extracellular matrix is generated, resulting in a histological appearance that is similar to native cartilage. Another advantage of the self-regenerating approach is that the cells are not constrained to a density or distribution based on scaffold structure. Immunohistochemical staining confirms that the collagen is predominantly type II rather than type I and is typical of hyaline articular cartilage. These values are calculated based on dry weight following lyophilization, and a comparison of wet and dry weights shows that dSRC contains higher amounts of water than native cartilage (Figure 1). This may be somewhat overestimated given that the dSRC samples are grown in media and are more difficult to dry compared to native cartilage prior to “wet” weighing.

While these results are encouraging, this study has some obvious caveats and limitations. It constitutes a model for how implanted dSRC may behave in vivo and should not be construed as a study to generate mechanically or chemically matched tissue prior to implantation. We observe hyaline maturation under our in vitro culture conditions, suggesting a minimum for what may be expected in situ in the knee joint in vivo. In fact, in an ongoing swine study, the dSRCs are implanted in lesions after only two weeks of maturation to ensure the physiological environment has an optimal influence on subsequent maturation. One limitation of this approach to cartilage regeneration in a clinical sense is the need to harvest autologous cells to generate the dSRC implant. In the clinical application, one may consider the use of stem cell progenitors or alternative sources such as auricular chondrocytes that would not involve the same level of invasive surgery to harvest. The SHEAR imaging of the tissue is performed with direct illumination on the laboratory bench to demonstrate its capability. However, there is no limitation to applying the SHEAR technology through an arthroscope, for example, which would also allow for minimally invasive evaluation of cartilage in healthy or diseased tissue in a longitudinal fashion.

In conclusion, this in vitro evaluation of the dSRC maturation process provides positive reinforcement in terms of both biochemical composition and mechanical performance. In this in vitro study, the physiological forces in the joint that can stress/stimulate regenerative tissue are lacking, but we are currently addressing this limitation in an ongoing in vivo study for swine cartilage regeneration.

## 4. Materials and Methods

### 4.1. Chondrocyte Isolation and dSRC Formation

Chondrocytes were isolated as previously described [20,21]. The cartilage was dissected, rinsed in PBS (Thermo Fisher Scientific, Waltham, MA, USA), and minced into small pieces using sterile blades. Cartilage was digested in 40 mL of Ham’s Nutrient Mixture F12 (Sigma-Aldrich, St. Louis, MO, USA) containing 0.1% collagenase type 2 (Worthington Biochemical, Freehold, NJ, USA) and 1% antimycin (Sigma-Aldrich) at 37 °C on a rocker (0.35 Hz) in a Heratherm IMH180 oven (Thermo Electron LED GmbH, Langenselbold, Germany) for 18 h. Following digestion, the contents were filtered through a 100 µm cell strainer (BD Biosciences, Bedford, MA, USA) into a 50 mL conical tube, followed by centrifugation (250 g, 15 min, 4 °C). The supernatant was aspirated and resuspended in chondrogenic media of Ham’s F-12 medium supplemented with 10% fetal bovine serum, 1% antibiotic/antimycotic solution, 1% nonessential amino acids, and 50 mg/mL ascorbic acid. All reagents were from Invitrogen, Carlsbad, CA, USA. The process was repeated twice, and following the final centrifugation, the cells were suspended in 10 mL of chondrogenic media for cell counting and validation of >90% viability, as described [21]. 

The dSRCs were formed as previously described [20,21]. Briefly, aliquots of 10 million chondrocytes were placed in sealed 15 mL conical polypropylene tubes and cultured for 14 days at 37 °C. The tubes were placed under continuous dynamic reciprocating motion at 40 cycles per minute. During this time, the chondrocytes aggregated and generated a pellet or sheet of new dSRC extracellular matrix. Media changes were performed every 3–4 days. The maturation of dSRC was evaluated periodically in vitro up to 12 weeks by evaluating biochemical composition, matrix morphological characteristics, and biomechanical performance. Native cartilage was obtained from freshly harvested swine knees using a 7 mm circular punch (Acuderm Acu-Punch Biopsy Punch, Fort Lauderdale, FL, USA). After harvesting full-thickness (cartilage + subchondral bone) specimens, the cores were reduced using a single-edge blade (AccuForge, Verona, VA, USA). Only the cartilage layer (3–4 mm) was used for the SHEAR, biochemical, and biomechanical analyses.

### 4.2. Biochemical Analysis

The biochemical composition of native cartilage and dSRC samples was quantified via colorimetric GAG and hydroxyproline assays [32]. Samples were first lyophilized for 24 h, with pre- and post-lyophilization weights measured to determine water content. Lyophilized samples were digested in papain solution [20 mg/mL papain, 100 mM sodium acetate, 10 mM EDTA, and 5 mM l-cysteine in 0.2 M sodium phosphate buffer (pH 6.4); all reagenmts from Sigma-Aldrich] at 65 °C for 24 h and used immediately for biochemical assays. The GAG content of the native and engineered cartilage samples was determined using the Blyscan^TM^ Sulfated Glycosaminoglycan (sGAG) Assay kit (BioColor, Carrickfergus, UK), whereby solubilized samples were incubated with dimethylene blue dye, the sGAG–dye complex was precipitated, the unbound dye was removed, and samples were then incubated with surfactant to dissociate the sGAG–dye complex. The absorbance of each sample was measured at 656 nm. A standard curve was generated using bovine tracheal chondroitin 4-sulfate provided in the kit.

The N-hydroxyproline content of each sample, commonly used as an indicator of collagen levels, was quantified using a Hydroxyproline Assay Kit (Sigma-Aldrich) in which lyophilized samples were first hydrolyzed then oxidized with chloramine T and the chromophore was then developed by reaction with 4-(dimethylamino)benzaldehyde (DMAB), which resulted in a colorimetric (560 nm) product. Standard curves were generated from authentic N-hydroxyproline (supplied in the kit) and soluble bovine collagen type I (Bico, Gothenburg, Sweden). Excellent agreement was seen between both standard curves based on an expected weight percentage of N-hydroxyproline in collagen of ~13%.

### 4.3. Laser Speckle rHEologicAl micRoscopy (SHEAR)

A significant development in this work was using SHEAR to measure the biomechanical properties of the chondrocyte-generated neocartilage. SHEAR is based on measuring the fluctuation of laser speckle, a granular pattern formed by the interference of light backscattered from the sample upon illumination by a laser beam [22,23,26,31]. This speckle fluctuation arises from the naturally occurring thermal (Brownian) motion of native tissue constituents, which is influenced by the mechanical “stiffness” of a sample, with the fluctuation rate being slower for stiffer materials. Specifically, the temporal fluctuation of laser speckle intensity is analyzed using the well-established diffusing wave spectroscopy formulation and the generalized Stokes–Einstein relation [33,34] to derive the shear viscoelastic modulus of the sample. The optical setup, working principle, and image reconstruction procedure of SHEAR were previously described in detail [22,26,31]. In brief, a 632 nm HeNe laser (HNL210LB, Thorlabs Inc., Newton, NJ, USA) was linearly polarized, expanded, and focused onto the back focal plane of a 10x 0.25 NA air objective (LMPLFLN10X, Olympus IE, Waltham, MA, USA) to illuminate the sample with a collimated beam. The optical power at the sample was 3.3 mW. Backscattered light was collected through the same objective lens via a beam splitter and acquired by a high-speed CMOS camera (acA2000–340km, Basler Inc., Exton, PA, USA). A speckle time series consisting of 1000 speckle frames over a region of interest of 290 μm × 580 μm was acquired at a frame rate of 250 Hz. The sample was laterally translated on a 2-axis motorized stage (MFA-CC, Newport Corporation, Franklin, MA, USA) to acquire speckle time series over a total area of 1.9 mm × 2.1 mm in each specimen. The optical resolution of the SHEAR microscope was 1.5 μm, supporting the spatial resolution of the reconstructed shear modulus map of approximately 15 μm after spatial averaging [31].

Native cartilage and dSRC samples were characterized as harvested with no additional processing. All measurements were conducted at room temperature. Native cartilage was measured with illumination of the articular surface. A total of 3 specimens were measured for each dSRC maturation time point and native cartilage. The magnitude of the shear modulus, |G*|, at 15 Hz is reported.

### 4.4. Shear Rheometry of Native Cartilage

To compare SHEAR to conventional rheometry, native cartilage samples harvested from the same swine knee were evaluated with a conventional oscillatory shear rheometer (AR-G2, TA Instruments, New Castle, DE, USA). The samples were harvested with a biopsy punch and sliced into a disk with a diameter of 8 mm and a thickness of 1 mm. A custom 8 mm diameter parallel plate with a serrated surface was used to prevent sample slippage. Due to the precise requirement of the sample geometry, rheometer evaluation of the self-generated dSRC matrix was not feasible. The top plate was lowered onto the sample until a normal force of 3 N was registered. A frequency sweep test over 0.5–40 Hz was performed at 23 °C with an oscillatory torque of 10 μN∙m (resulting in <0.2% strain) under normal force control. A total of 4 samples were measured. The magnitude of the shear modulus at the frequency of 15 Hz is reported.

### 4.5. Histology

After 2, 4, 8, and 12 weeks of in vitro culture, the dSRC samples were embedded in paraffin, and 5 µm sections were stained with hematoxylin and eosin (H&E) to evaluate neomatrix composition. Masson’s Trichrome staining was performed to assess the collagen content of the neomatrix qualitatively. Safranin O (sulfated glycosaminoglycans [GAGs]), Toluidine blue (proteoglycan), and Masson’s Trichrome (collagen) staining were also performed to further evaluate the biochemical composition of the neomatrix. The slides were scanned and imaged by NanoZoomer 2.0HT Digital Pathology (Hamamatsu Photonics K.K., Shizuoka, Japan) and analyzed using NDP.View2 software (Version 2.9, Hamamatsu Photonics K.K.).

### 4.6. Immunohistochemistry

Immunohistochemical staining for collagen types I (fibrocartilage) and II (hyaline cartilage) was performed on 12-week samples. An anti-collagen I antibody (#AB6308, Abcam, Cambridge, MA, USA) and an anti-collagen type II antibody (#AB34712, Abcam) were used. Antibodies were diluted 1:500 in 10% goat serum (Invitrogen) and 3% bovine serum albumin-blocking buffer (Sigma Aldrich, St. Louis, MO, USA) before use. Target Retrieval Solution (DAKO, Glostrup, Denmark #S1699, pH 6.0–6.2) was used for antigen retrieval. Slides were left for 30 min in the blocking buffer and then incubated with the primary antibody for 60 min. Sections where the incubation with the primary antibodies was suppressed were used as negative controls. The slides were washed extensively in PBS followed by incubation for 30 min with Mach 2 rabbit AP-Polymer secondary antibody (DAKO #K401011-2), and washed again in PBS (3 × 5 min). Finally, slides were washed in distilled water, and the sections were probed with AlexaFluor 555-conjugated secondary antibodies (1:200, Life Technologies, Carlsbad, CA, USA) and counterstained with 4′, 6-diamidino-2-phenylindole dihydrochloride (DAPI) to reveal cell nuclei. All samples were processed at the same time to minimize sample-to-sample variation. Fluorescent images were acquired using the NanoZoomer whole-slide scanner described above.

### 4.7. Statistical Analysis

Biochemical results between the dSRC and control groups were presented as mean ± standard deviation (SD). Statistical significance was determined by Student’s *t*-test, with a statistically significant difference set at *p* < 0.05 in all statistical tests. For biomechanical analysis, values of |G*| were spatially mapped in all 3 samples for each of the dSRC maturation times, and the native cartilage was combined for statistical comparison between any pair of categories. Statistical significance was determined by the Wilcoxon-type nonparametric Kruskal–Wallis test with a significant difference in chi-squared statistics on the group-adjusted (Bonferroni correction for multiple comparisons among groups) two-sided pairwise comparison set at *p* < 0.05. Statistical analysis was conducted with MATLAB 2022a.

## Data Availability

The raw data supporting the conclusions of this article will be made available by the authors on request.

## Abbreviations

The following abbreviations are used in this manuscript:

ACI	Autologous chondrocyte implantation
CMOS	Complementary metal-oxide semiconductor
DAPI	4′,6-Diamidino-2-phenylindole dihydrochloride
DMAB	4-(dimethylamino)benzaldehyde
dSRC	Dynamic self-regenerating cartilage
|G*|	Shear modulus
GAG	Glycosaminoglycan
H&E	Hematoxylin and eosin
HeNe	Helium neon
OA	Osteoarthritis
PEG	Polyethylene glycol
sGAG	Sulfonated glycosaminoglycan
SD	Standard deviation
SHEAR	Speckle rHEologicAl micRoscopy
